# Overexpression of the SARS-CoV-2 receptor angiotensin converting enzyme 2 in cardiomyocytes of failing hearts

**DOI:** 10.1038/s41598-022-04956-y

**Published:** 2022-01-19

**Authors:** Kristina Vukusic, Annika Thorsell, Aida Muslimovic, Marianne Jonsson, Göran Dellgren, Anders Lindahl, Joakim Sandstedt, Ola Hammarsten

**Affiliations:** 1 Department of Laboratory Medicine, Institute of Biomedicine, Sahlgrenska Academy, University of Gothenburg, Sahlgrenska University Hospital, Bruna stråket 16, 41345 Gothenburg, Sweden; 2 Department of Clinical Chemistry, Sahlgrenska University Hospital, Gothenburg, Sweden; 3 Proteomics Core Facility at Sahlgrenska Academy, Gothenburg University, Gothenburg, Sweden; 4 Department of Cardiothoracic Surgery, Sahlgrenska University Hospital, Gothenburg, Sweden; 5 Department of Molecular and Clinical Medicine, Institute of Medicine, Sahlgrenska Academy, University of Gothenburg, Gothenburg, Sweden

**Keywords:** Biochemistry, Biotechnology, Cell biology, Immunology, Molecular biology, Physiology, Biomarkers, Cardiology, Diseases, Medical research, Molecular medicine

## Abstract

Hospitalized patients who die from Covid-19 often have pre-existing heart disease. The SARS-CoV-2 virus is dependent on the ACE2 receptor to be able to infect cells. It is possible that the strong link between cardiovascular comorbidities and a poor outcome following a SARS-CoV-2 infection is sometimes due to viral myocarditis. The aim was to examine the expression of ACE2 in normal hearts and hearts from patients with terminal heart failure. The ACE2 expression was measured by global quantitative proteomics and RT-qPCR in left ventricular (LV) tissue from explanted hearts. Immunohistochemistry was used to examine ACE2 expression in cardiomyocytes, fibroblasts and endothelial cells. In total, tissue from 14 organ donors and 11 patients with terminal heart failure were included. ACE2 expression was 2.6 times higher in 4 hearts from patients with terminal heart failure compared with 6 healthy donor hearts. The results were confirmed by immunohistochemistry where more than half of cardiomyocytes or fibroblasts showed expression of ACE2 in hearts from patients with terminal heart failure. In healthy donor hearts ACE2 was not expressed or found in few fibroblasts. A small subpopulation of endothelial cells expressed ACE2 in both groups. Upregulated ACE2 expression in cardiomyocytes may increase the risk of SARS-CoV-2 myocarditis in patients with heart failure.

## Introduction

Cardiovascular complications are common in COVID-19 infection. Pre-existing heart disease and higher levels of the cardiac damage biomarker troponin during infection predict a poor prognosis^1–5^. Recently, a report from the Swedish Registry for Cardiopulmonary Resuscitation showed higher mortality from acute cardiac arrest among COVID-19 patients^6^. The mechanisms behind the effect of a SARS-CoV-2 infection on patients with cardiovascular comorbidities remain incompletely understood but damage due to viral endocarditis remains a possibility, as indicated by isolated case reports^7^.

Like SARS-CoV-1, SARS-CoV-2 infects the host using the membrane-bound angiotensin-converting enzyme 2 (ACE2) as one of its receptors^8^. ACE2 is expressed in alveolar epithelial cells but also in the heart, intestine, kidneys, brain, testes and liver^9^. In addition, the ACE-2 receptor is also expressed in endothelial cells and direct viral replication in vessels could be the reason behind the thromboembolic events during COVID-19 infection and the need for heparin treatment^10^.

Among its many functions, ACE-2 also acts as a regulator in hypertension, heart failure and myocardial infarction^11,12^. SARS-CoV-2 has greater affinity for human ACE-2 than SARS-Cov1 and it has been speculated that this could result in viral replication in multiple organs systems during the COVID-19 infection^13,14^. In line with this, overexpression of ACE-2 in a mouse model enhanced disease severity^15^.

In the present study, we investigate the expression of ACE2 in a healthy and failing human myocardium and correlate the expression to biomarkers of fibroblasts, cardiomyocytes and endothelial cells to identify a possible cardiac cell target for the SARS-Cov-2 virus invasion.

## Materials and methods

### Human cardiac biopsies

The study was approved by the Research Ethics Board at the Sahlgrenska Academy, University of Gothenburg, Sweden, following the Helsinki Declaration. This study was based on whole explanted hearts from which biopsies were obtained from the anterolateral wall of the left ventricle (LV).

Two groups of research subjects were included through collaboration with the Transplant Institute at Sahlgrenska University Hospital. In the first group, cardiac tissue from 13 multi-organ donors was obtained. The hearts were not suitable for heart transplantation but explanted for homograft procurement and used in the present study after the valves were harvested. Organ donors with chronic heart failure were excluded. All had documentation of consent from the donor, stating that their organs could be used for other medical purposes than organ donation. The second group included 11 patients with severe heart failure undergoing cardiac transplantation. After signed informed consent was obtained, cardiac tissue was harvested from the hearts that were removed during cardiac transplantation surgery. The clinical background is summarized in Tables 1 and 2.

**Table 1:** Clinical background of included multi-organ donors.

Donor	Sex	Age	Cause of death	Other diseases	Tissue used for
1	F	19	Ischemic cerebral edema due to cardiac arrest	Anorexia	Proteomics IHC PCR
2	F	43	Ischemic cerebral edema due to cardiac arrest caused by major bleeding	None	Proteomics IHC PCR
3	M	19	Ischemic cerebral edema due to cardiac arrest	HF in the acute setting	Proteomics IHC PCR
4	M	21	Ischemic cerebral edema, due to suicide	HF in the acute setting	Proteomics IHC PCR
5	M	46	Ischemic cerebral edema, due to suicide	None	Proteomics IHC PCR
6	F	63	Ischemic cerebral edema, due to cardiac arrest	Ischemic heart disease, hypertension, obesity, hypothyroidism, diabetes type 2, renal insufficiency, emphysema	Proteomics IHC PCR
7	F	44	Ischemic cerebral edema, due to suicide	Psychiatric disease, Mitral valve insufficiency	IHC PCR
8	M	74	Intracerebral hemorrhage	Previous stroke	PCR
9	M	62	Subarachnoid hemorrhage	Atrial fibrillation, previous Maze surgery	PCR
10	M	51	Ischemic cerebral edema due to cardiac arrest	HF in the acute setting	PCR
11	F	42	Intracerebral hemorrhage	Takotsubo cardiomyopathy in the acute setting	PCR
12	M	52	Cardiac arrest	HF in the acute setting	PCR
13	F	75	Intracerebral hemorrhage	Atrial Fibrillation, ischemic heart disease, previous AMI	PCR
14	F	50	Intracerebral hemorrhage	Previous ventricular tachycardia, suspected previous AMI, suspected Takotsubo cardiomyopathy in the acute setting	PCR

Table summarizes the clinical background of the organ donors not suitable for the cardiac transplantation. F = female, M = Male, AMI = Acute Myocardial Infarction, HF = Heart Failure, Proteomics = Global Quantitative proteomics, IHC = Immunohistochemistry, PCR = Polymerase chain reaction.

**Table 2:** Clinical background of patients with terminal heart failure.

Patient	Sex	Age	Cause of heart failure	NYHA	Assist	Other diseases	Tissue used for
1	M	65	Ischemic dilated cardiomyopathy	IIIA	No	ASD, atrial fibrillation and asthma	Proteomics IHC PCR
2	F	60	Restrictive cardiomyopathy, due to amyloidosis	IIIA	No	PAH, asthma, amyloidosis and myeloma	Proteomics IHC PCR
3	F	64	Idiopathic dilated cardiomyopathy	IIIB	No	PAH, valvular disease, atrial fibrillation, diabetes type II, hypertension, renal insufficiency, hypothyreosis and previous ovarian cancer	Proteomics IHC PCR
4	M	27	Idiopathic dilated cardiomyopathy	III	Yes	PAH, atrial fibrillation	Proteomics PCR
5	M	66	Idiopathic dilated cardiomyopathy	IIIA	Yes	PAH, previous stroke and diabetes type II	IHC PCR
6	M	67	Ischemic cardiomyopathy	IIIA	Yes	PAH, previous AMI, atrial fibrillation and renal insufficiency	IHC PCR
7	M	45	Hypertrophic cardiomyopathy	IIIB	No	PAH	IHC PCR
8	M	39	Ischemic cardiomyopathy	III	No	PAH, previous AMI, valvular disease, diabetes type I, mild retinopathy and nephropathy	PCR
9	M	66	Dilated cardiomyopathy due to amyloidosis	IIIB	No	Diabetes type II and PAH	PCR
10	M	50	Ischemic dilated cardiomyopathy	IIIA	No	Diabetes type II, hypertension, and previous stroke	PCR
11	M	61	Hypertrophic cardiomyopathy	III	No	Previous stroke, previous atrial fibrillation, previous Maze surgery, renal insufficiency, COPD	PCR

Table summarizes the clinical background of the included patients that underwent cardiac transplantation due to heart failure. F = female, M = Male, NYHA = New York Heart Association, PAH = Pulmonary Arterial Hypertension, AMI = Acute Myocardial Infarction, ASD = Atrial Septum Deficiency, COPD = Chronic Obstructive Pulmonary Disease, Assist = mechanical assist (i.e. LVAD / BiVAD), LVAD = Left Ventricular Assist Device, Proteomics = Global Quantitative proteomics, IHC = Immunohistochemistry, PCR = Polymerase chain reaction.

### Relative protein quantification

Tissue from six organ donors (age 19–63 years) and from four patients (age 28–65 years) in the heart failure group was used. The tissue was snap-frozen and stored at −80 °C until use.

Proteins were extracted using a lysis buffer (50 mM triethylammonium bicarbonate (TEAB), 2% sodium dodecyl sulfate (SDS)). 30 μg from each sample and a reference sample were digested into peptides using filter-aided sample preparation (FASP)^16^. The reference consisted of aliquots from each group to provide representative references. Peptides were labeled using TMT 11-plex isobaric mass tagging reagents (Thermo Fisher Scientific), according to the manufacturer’s instructions. The TMT set was fractionated using basic reverse phase liquid chromatography (pH 10) into 20 fractions and analyzed in an Orbitrap Fusion Tribrid mass spectrometer interfaced with the Easy-nLC1200 liquid chromatography system (Thermo Fisher Scientific). The peptides were separated on an analytical C18 column using a gradient from 4 to 28% acetonitrile in 0.2% formic acid over 75 min. Relative quantification was performed using Proteome Discoverer version 2.4 (Thermo Fisher Scientific) and the Mascot search engine (v. 2.5.1 Matrix Science, London, UK) matching against the SwissProt *H. sapiens* database (July 2019). Peptide and fragment tolerances were set to 5 ppm and 0.6 Da, zero missed cleavages, variable methionine oxidation, fixed cysteine methylthiolation and TMT-6 modifications on lysine and peptide N-termini. Percolator was used for PSM validation at the 1% FDR threshold. TMT reporter ions were identified in the MS3 HCD spectra with a 3 mmu mass tolerance, and samples were normalized on the total peptide amount. The reference sample was the denominator and used to calculate abundance ratios. Statistical analysis was performed with Perseus software^17^ (version 1.6.10.45), two-side students t-test on the Log2 protein abundance ratios for *p* value calculations. For the volcano plot the number of randomization was set to 250, FDR to 0.05 and S0 to 0.1.

### RNA isolation and gene expression by qPCR

Left ventricular tissue was obtained from 14 organ donors (age 19–74 years) and 11 patients with severe heart failure in conjunction with heart transplantation (age 27–67 years). The tissue was preserved in RNAlater. Total RNA was extracted using the reagents and equipment from Qiagen. Briefly, the tissue was homogenized with TissueLyser LT and Qiazol and then purified using a RNeasy Mini column with DNase1 treatment for removal of residual genomic DNA. The cDNA was prepared from total RNA using a High-Capacity cDNA reverse transcription kit with RNase Inhibitor #4374967 and TaqMan Gene Expression Master Mix #4369542 (Applied Biosystems).

The human TaqMan gene expression assay *ACE2* Hs01085333_m1 was used for the gene of interest, and *PPIA* Hs99999904_m1 as the reference gene. The relative comparative method was used to analyze the RT-qPCR data (Sequence Detector User Bulletin 2, Applied Biosystems) and the relative quantification (RQ) values were calculated using *PPIA* as the reference gene and an in-house calibrator sample. Gene expression data are presented in relative units.

### Immunohistochemistry

Biopsies from the LV from seven organ donors and six heart failure patients were embedded in Tragacant mounting medium (Histolab Products AB, Gothenburg, Sweden), frozen in liquid nitrogen and stored at −80 °C. The frozen tissues were sectioned into 7 μm serial sections that were fixed in −20 °C acetone for 10 min and washed in Phosphate Buffer Saline (PBS). A 30-min blocking step followed, with 2% bovine serum albumin, 0.3% Triton–X100 and 5% goat serum (Invitrogen, Carlsbad, CA, USA) diluted in PBS.

Primary antibodies were diluted according to Table 3, added to the sections and incubated at 4 °C overnight in a humidified chamber. Results were visualized by staining with secondary antibodies: *goat anti-rabbit Alexa Fluor 546 or goat anti-mouse Alexa Fluor 647* (Invitrogen) for 1–2 h at RT. To enable triple staining, cTnT antibody was conjugated with Alexa 488 using the Zenon kit (Invitrogen) and a 1:6 molar ratio. After incubation with secondary antibodies, the samples were washed and incubated with the Zenon conjugated cTnT antibody for another hour. Sections were fixed with Histofix (Histolab) for 15 min and mounted with Prolong Gold Antifade reagent with DAPI (Invitrogen). Corresponding isotype controls for the primary antibodies were used for determining the background and did not show any specific staining.

**Table 3:** Antibodies used for immunohistochemistry.

Biomarker	Primary antibody	Concentration (mg/ml)	Dilution	Company	Catalog number
ACE2	Rabbit polyclonal IgG	1.0	1:100	Abcam	ab15348
cTnT	Mouse monoclonal IgG1	0.2	1:50	Thermo Fisher	MS-295-P1
CD31	Mouse monoclonal IgG1	0.5	1:100	Bio Legend	303,102
TE7	Mouse monoclonal IgG1	0.2	1:300	Santa Cruz	sc-73603

Thermo Fisher = Thermo Fisher Scientific, Santa Cruz = Santa Cruz Biotechnology.

### Image analysis and quantification

The results were visualized using an ECLIPSE Ti inverted microscope (Nikon Corporation, Tokyo, Japan). A Nikon DS-2Mv camera was used for brightfield histology images. For analyses of immunohistochemistry, fluorescence images were acquired with an Andor Zyla camera. Large images: 7 × 7 fields shot with the 20× objective were scanned at three Z levels to capture all parts of the large images in focus. Generally, four channels (DAPI, Alexa 488, Alexa 546 and Alexa 647) were acquired.

All images were exported to Image J software (v. 1.47 h, Fiji distribution)^18^ for further analysis. For each channel, displayed pixel ranges were set so that most of the background was extinguished. Isotypic controls were treated in the same way. The composite photos were used for analysis of the expression of biomarkers. Two large images, composed of stitched photos of 49 fields by 20× objective were analyzed for each individual.

For quantification of ACE2 expression, an intensity threshold was first set for each staining using a customized plugin, to reduce background staining. Pixel values below the threshold were set to zero. The threshold was then subtracted from pixel values above the threshold in order to get a continuous distribution of pixel values. All images were treated equally. The threshold level was set based on background staining of isotype control images. Areas of quantification were created include most of the stained tissue, but excluding obvious artefacts and parts of the images not in focus (Suppl. Figure 1). Mean pixel intensity measurements were carried out on ACE2 expression for each image. Outliers were excluded based on the 1.5 interquartile range (IQR) method. In total, measurements of 3 images were regarded as outliers (two donors, one heart failure subject), and were excluded. In each case, the other replicate image was not regarded as outlier. After outlier exclusion, a mean pixel intensity was calculated for each study subject. Difference in ACE2 expression between donors and heart failure subjects were tested using Wilcoxon rank sum test. *P* < 0.05 was considered significant. All statistical calculations were carried out using R v. 4.0.2 (R Core Team 2020 https://www.R-project.org/).

## Results

### Histology

**Figure 1:**
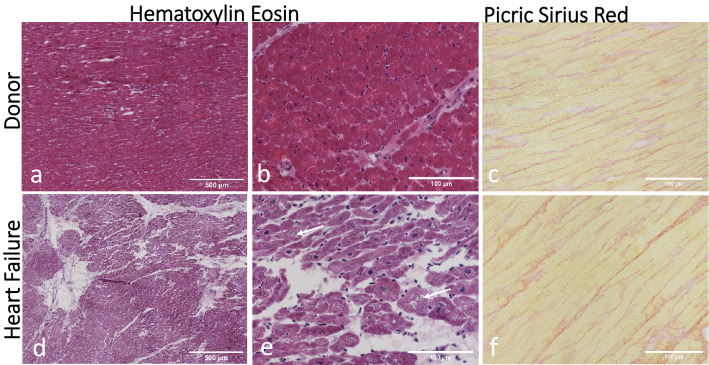
Histology of the left ventricular myocardium. (**a, b**) Hematoxylin–Eosin staining of tissue from a representative organ donor displays the histology of a normal myocardium. **(c)** Picric Sirius red staining shows the network of collagen fibers in red between cardiomyocytes. **(d, e)** A representative image of tissue from the heart failure group displaying excessive fibroses, hypertrophic cardiomyocytes and irregular nuclei (arrows). **(f)** Collagen fibers in red between hypertrophic cardiomyocytes.

Tissue sections were stained with Hematoxylin Eosin and Picric Sirius red for histology examination (Fig. 1). LV tissue from the organ donor group displayed a normal myocardium histology. Tissue from the Heart Failure (HF) group showed histopathological changes, including excessive fibrosis, infiltration of adipocytes and hypertrophy of cardiomyocytes. Cardiomyocyte nuclei were enlarged and irregular, often with lipofuscin accumulation.

### Relative protein quantification

**Figure 2:**
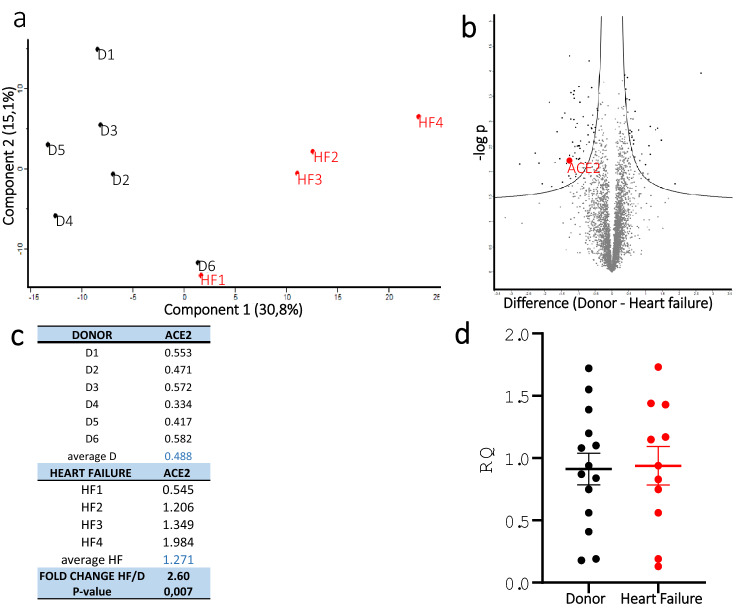
Quantitative proteomics and gene expression of ACE2. **(a)** Principal component analysis (PCA) of the D = Donor and HF = Heart Failure groups based their proteomic expression files. The first and second components segregate the groups and account for 30.8 and 15.1% of the variability, respectively. **(b)** Vulcano plot of al included proteins showing that ACE2 was one of the proteins that was overexpressed in the HF group. **(c)** Abundance ratios from TMT quantification where sample abundances are divided with reference abundance. ACE2 was upregulated 2.6 × with a *p* value of 0.007 (Two-sided student t test) in the HF group. **(d) RT-qPCR** relative quantification of the gene expression of *ACE2* based on RNA from left ventricular tissue from 14 organ donors and 11 HF patients. There was no difference in ACE2 gene expression between the groups.

We analyzed tissue from six organ donors and four patients from the HF group, using global quantitative proteomics, for relative quantification of proteins between samples. A total of 5989 proteins were quantified and among these, 669 proteins were statistically differentially expressed between the two groups (*p* value < 0.05). For an overall assessment of the proteomics of the two groups, we applied principal component analysis (PCA) to all expressed proteins (Fig. 2a). Principal component 1 (PC1) clearly separates the Donor and HF groups where the individuals within the groups cluster together. Donor six, 63 years old, with cardiovascular disease, diabetes and obesity (Table 1), reflects the biological variability among the organ donors and is an outlier compared with the other five healthy donors. ACE2 was one of the major contributors to the segregation of the two groups. Vulcano plot of al included proteins shows that ACE2 was one of the top 20 proteins that was overexpressed in the HF group (Fig. 2b) and was upregulated 2.6 times (Fig. 2c). Proteases TMPRSS2 and CTSL, relevant for SARS-CoV-2 virus entry into the cell were not detected (data not shown).

### Gene expression

Relative ACE2 mRNA levels were quantified using RT-qPCR and performed on individual tissue samples from 14 organ donors and 11 patients from the HF group. The results showed no difference in ACE2 mRNA levels between the groups (Fig. 2d).

### Immunohistochemistry

**Figure 3:**
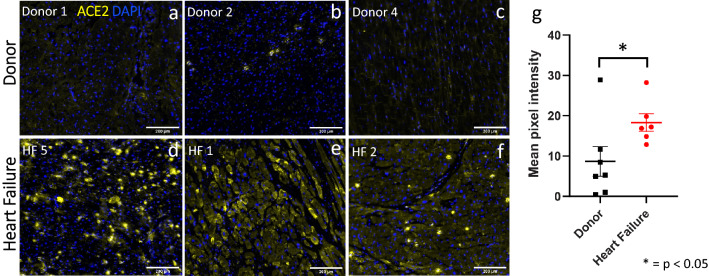
Protein expression of ACE2 by Immunohistochemistry (IHC). **(a-c)** Representative images of IHC staining of tissue sections from three organ donors showing no or few ACE2-expressing cells. **(d-e)** Representative images from three HF patients showing ACE2-expressing cells. **(g**) Measurement of mean pixel intensity show that heart failure group had a significant higher ACE2 staining intensity compared to the donor group (*p* = 0.035).

**Figure 4:**
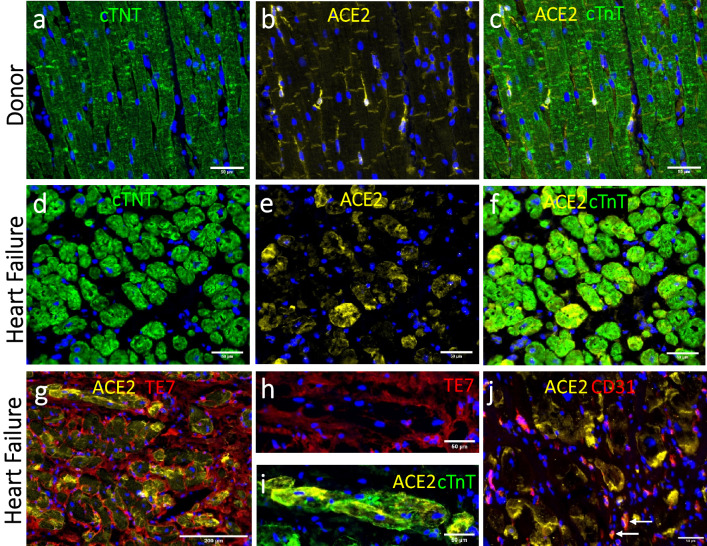
Protein expression of ACE2 in different cell phenotypes. **(a-c)** Representative images of IHC staining of the myocardium from an organ donor showing ACE2 expressing cells between cTnT + cardiomyocytes. ACE2 staining did not overlap with the expression of cTnT. **(d-f)** cTnT + /ACE2 + cells were identified in the myocardium from three individuals with terminal heart failure, showing ACE2 expression in cardiomyocytes. **(g)** In the HF group, most of the ACE2 + cells were cardiomyocytes. **(h, i)** Enlargement of ACE2 + /cTnT + cardiomyocytes surrounded by the Te7 + fibroblasts. **(j)** For the most part, ACE2 staining did not overlap with the CD31 endothelial staining in either of the groups. ACE2 + /CD31 + cells could be found (arrows), (see Suppl. Figure 3 for more details).

LV tissue sections from seven organ donors and six patients with terminal HF were analyzed for protein expression of ACE2 in different cell types. ACE2 antibody was combined with antibodies detecting the most common cardiac lineage markers; cTnT staining cardiomyocytes, CD31 for endothelial cells and TE-7 for fibroblasts. Tissue from three donors were negative for ACE2 expression while a few positive cells were detected in tissue from four donors (Fig. 3a-c). Donor six, the outlier with higher age and cardiovascular disease, displayed a few more ACE2 + cells (Suppl. Figure 2). In contrast, ACE2 + cells were detected in tissue from all six HF patients (Fig. 3d-f). When ACE2 expression in the images was quantified, the heart failure group had a significant higher expression compared to the Donor group (Fig. 3g). Cardiomyocytes in tissue from donor hearts did not express ACE2 since no co-expression of the cTnT and ACE2 was observed. The few ACE2 + cells were found between cTnT + cardiomyocytes (Fig. 4a-c).

In the HF myocardium, many cTnT + /ACE2 + cells were identified in three of the patients (Table 2; ID2, 3 and 7) showing ACE2 expression in more than half of cardiomyocytes (Fig. 4d-f). cTnT-/ACE2 + cells were detected in the tissue from the other three HF patients (Table 2; ID 1,5 and 6), suggesting another cell phenotype (Suppl. Figure 3a–c). These cells were ACE2 + /TE7 + fibroblasts (Suppl. Figure 3d–f). The few ACE2 + cells in the organ donor group were TE7 + fibroblasts (Suppl. Figure 4). In the HF group, ACE2 expression was found in cardiomyocytes with cTnT + /ACE2 + /TE7- signature (Fig. 4g-i and Suppl. Figure 4).

For the most part, ACE2 and CD31 staining did not overlap in either of the groups (Suppl. Figure 5). However, a small subpopulation of CD31 + endothelial cells that did express ACE2 could be found (Fig. 4j arrows).

## Discussion

We found that ACE2 levels were roughly three times higher and that ACE2 was one of the top 20 proteins that were relatively overexpressed in terminal heart failure patients that underwent heart transplantation. The relative overexpression was found in cardiomyocytes and fibroblasts. Upregulation of the SARS-CoV-2 virus receptor ACE2 could result in a greater likelihood of myocarditis and mortality in patients with heart failure, a possibility that has not been explored. Using immunohistochemistry, we confirmed a higher expression level of ACE2 in heart failure subjects compared to donors. Two different expression patterns were observed, where ACE2 was expressed by cTnT + cardiomyocytes in the myocardium from three of the heart failure patients or by TE7 + fibroblasts in the other three patients. Different underlying diseases behind the heart failure might explain the expression of ACE2 in fibroblasts or cardiomyocytes. Clinical background of the included patients did not provide any clear answers to evaluate this question further.

Several previous studies have shown that ACE2 is expressed in the cardiovascular system. Using proteomics, Wicik et al.^19^ found similar ACE2 expression levels in the respiratory and cardiovascular systems, supporting the theory that heart tissue is a potential target of SARS-CoV-2.

In the present study, the ACE2 mRNA levels showed no difference between healthy myocardium and heart failure in line with a previous study by Battle et al.^20^. In contrast increased mRNA levels of ACE2 in failing hearts were reported by Goulter et al.^21^. This discrepancy between our results remains unexplained. Notably, in the study by Goulter the control group consisted of donor hearts without chronic heart failure similarly to ours, whereas in the study by Battle limited data on the clinical background was provided. It could be hypothesized that differences in clinical background and medication could account for the discrepancies between studies.

Previous studies indicate that endothelial cells express ACE2 in many organs^22^ as well as the heart^23,24^. The tendency for thromboembolic conditions and edema to be induced in severe COVID-19 has been ascribed to viral infections and the destruction of endothelial cells. Evidence of direct viral infection of the endothelial cells and diffuse endothelial inflammation was reported in COVID-19 patients. Varga et al.^25^ therefore suggest that SARS-CoV-2 infection induces endothelialitis in several organs. Our results show that most of the high ACE2 expression in the terminal heart failure was located in cardiomyocytes or fibroblasts. Most of the CD31 + cells did not express ACE2 even if a small subpopulation of CD31 + /ACE2 + cells was found showing expression in endothelial cells and possibly pericytes. Previously, myocardium was examined and ACE2 expression found in macrophages, endothelium and myocytes^24^ and in line with our results, qualitatively increased expression of ACE2 was found in heart failure. However, these studies^22,24^ used light microscopy and histology for detection ACE2 but no co-staining with specific biomarkers was performed.

It should be acknowledged that global quantitative proteomics may not detect less abundantly expressed proteins. This might be an explanation for why we did not detect proteases TMPRSS2 nor CTSL, important for the SARS-CoV-2 virus entry into the cell. In line with our results Liu et al.^26^ reported far higher ACE2 mRNA levels in human heart with low expression of TMPRSS2.

In conclusion, we report three times greater expression of the COVID-19 receptor in heart failure. Most of the ACE2 + cells co-expressed Troponin T, indicating that cardiomyocytes are possible target of the SARS-CoV-2 virus. The other cell type expressing ACE2 are the cardiac fibroblasts. This might explain the involvement of the myocardium in COVID-19 and the high mortality among patients with cardiovascular comorbidities.

### Ethics approval

The study was approved by the Research Ethics Board at the Sahlgrenska Academy, University of Gothenburg, Sweden, following the Helsinki Declaration.

### Consent to participate

Documentation of consent from the multi organ donors, stating that their organs could be used for other medical purposes than organ donation. Signed informed consent was obtained from heart failure patients before cardiac transplantation surgery.

## Supplementary Information

Supplementary Figures.
