# Supplementary material for: Overexpression of the SARS-CoV-2 receptor angiotensin converting enzyme 2 in cardiomyocytes of failing hearts
Source: Sci Rep. 2022 Jan 19;12:965. doi: 10.1038/s41598-022-04956-y (PMC8770525; doi:10.1038/s41598-022-04956-y)
Supplement: Supplementary file 1 — Supplementary Figures. [file 41598_2022_4956_MOESM1_ESM.pptx]

## Slide 1
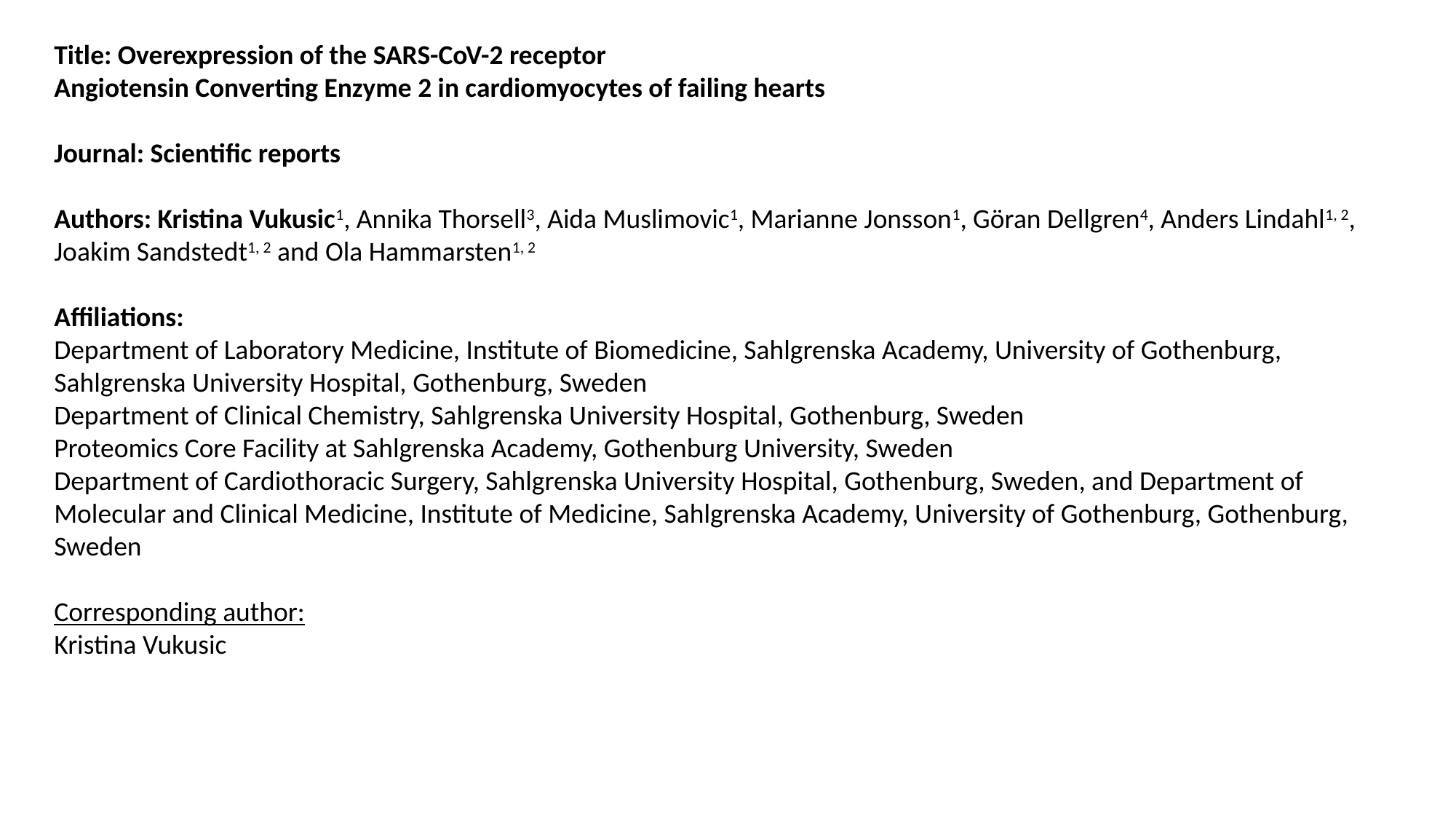

Title: Overexpression of the SARS-CoV-2 receptor
Angiotensin Converting Enzyme 2 in cardiomyocytes of failing hearts
Journal: Scientific reports
Authors: Kristina Vukusic1, Annika Thorsell3, Aida Muslimovic1, Marianne Jonsson1, Göran Dellgren4, Anders Lindahl1, 2, Joakim Sandstedt1, 2 and Ola Hammarsten1, 2
Affiliations:
Department of Laboratory Medicine, Institute of Biomedicine, Sahlgrenska Academy, University of Gothenburg, Sahlgrenska University Hospital, Gothenburg, Sweden
Department of Clinical Chemistry, Sahlgrenska University Hospital, Gothenburg, Sweden
Proteomics Core Facility at Sahlgrenska Academy, Gothenburg University, Sweden
Department of Cardiothoracic Surgery, Sahlgrenska University Hospital, Gothenburg, Sweden, and Department of Molecular and Clinical Medicine, Institute of Medicine, Sahlgrenska Academy, University of Gothenburg, Gothenburg, Sweden
Corresponding author:
Kristina Vukusic

## Slide 2
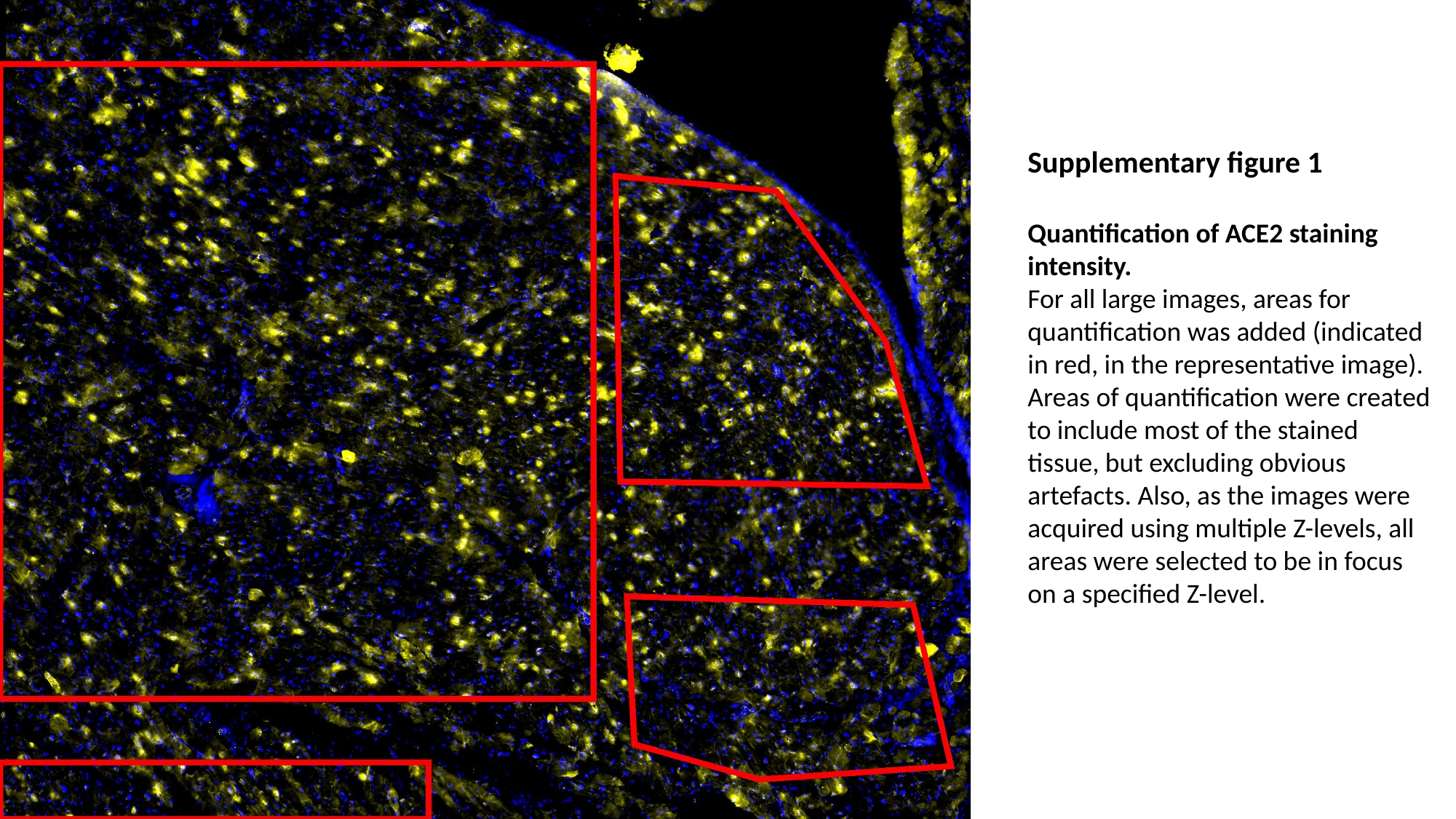

Supplementary figure 1
Quantification of ACE2 staining intensity.
For all large images, areas for quantification was added (indicated in red, in the representative image). Areas of quantification were created to include most of the stained tissue, but excluding obvious artefacts. Also, as the images were acquired using multiple Z-levels, all areas were selected to be in focus on a specified Z-level.

## Slide 3
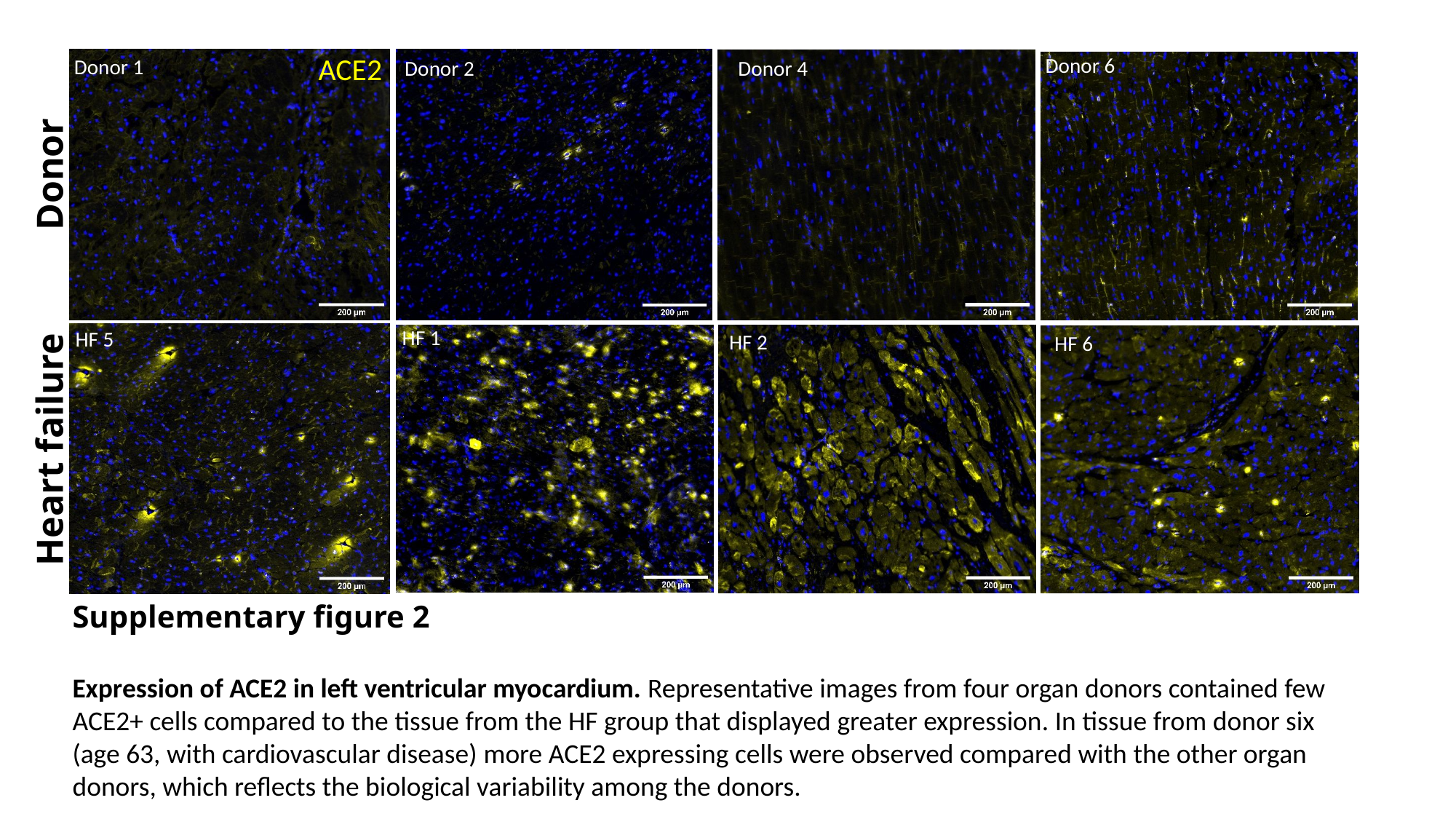

ACE2
Donor 6
Donor 1
Donor 2
Donor 4
Donor
HF 1
HF 5
HF 2
HF 6
Heart failure
Supplementary figure 2
Expression of ACE2 in left ventricular myocardium. Representative images from four organ donors contained few ACE2+ cells compared to the tissue from the HF group that displayed greater expression. In tissue from donor six (age 63, with cardiovascular disease) more ACE2 expressing cells were observed compared with the other organ donors, which reflects the biological variability among the donors.

## Slide 4
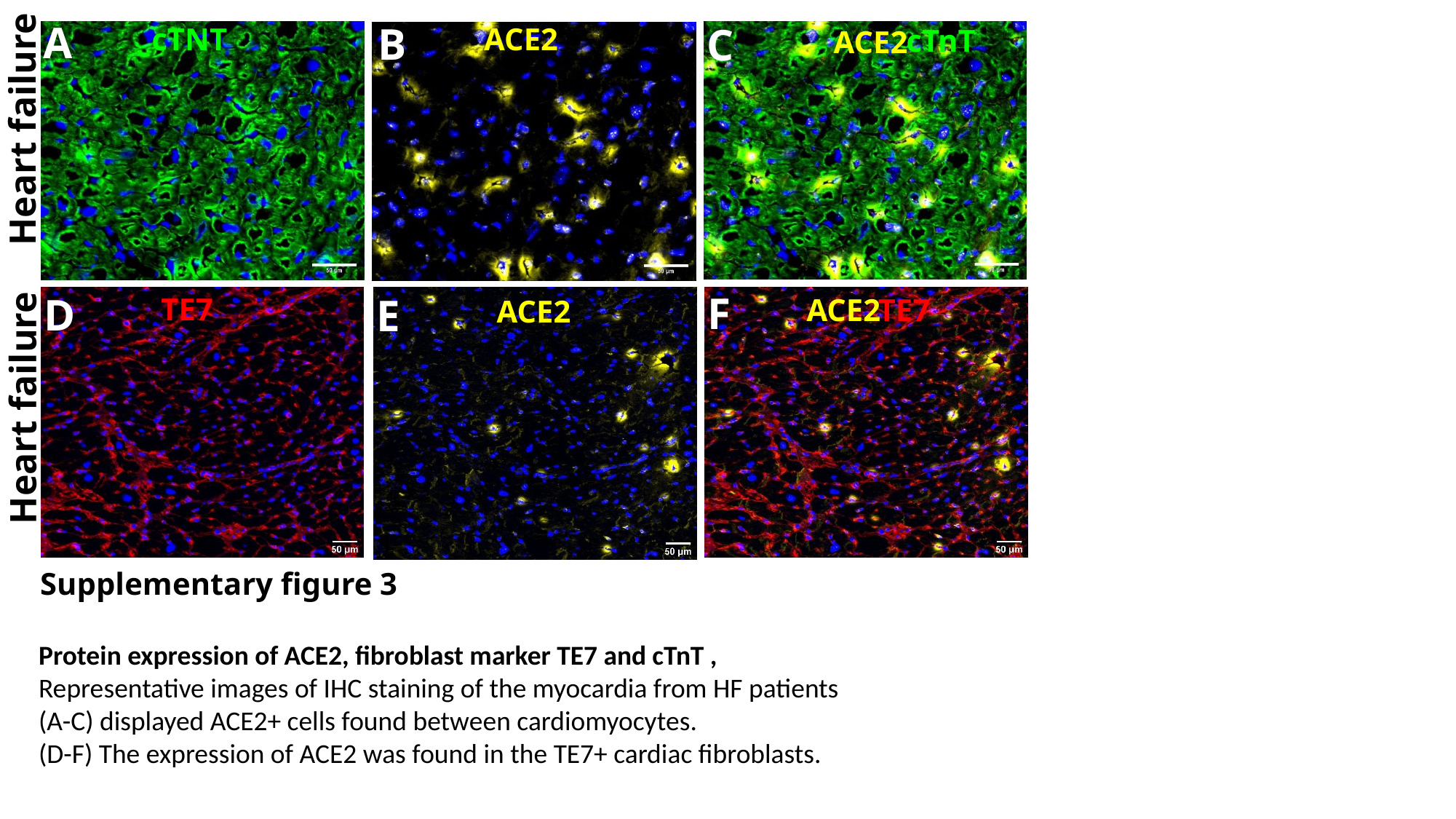

A
B
C
cTNT
ACE2
cTnT
ACE2
Heart failure
E
D
F
F
D
E
TE7
TE7
ACE2
ACE2
Heart failure
 Supplementary figure 3
 Protein expression of ACE2, fibroblast marker TE7 and cTnT ,
 Representative images of IHC staining of the myocardia from HF patients
 (A-C) displayed ACE2+ cells found between cardiomyocytes.
 (D-F) The expression of ACE2 was found in the TE7+ cardiac fibroblasts.

## Slide 5
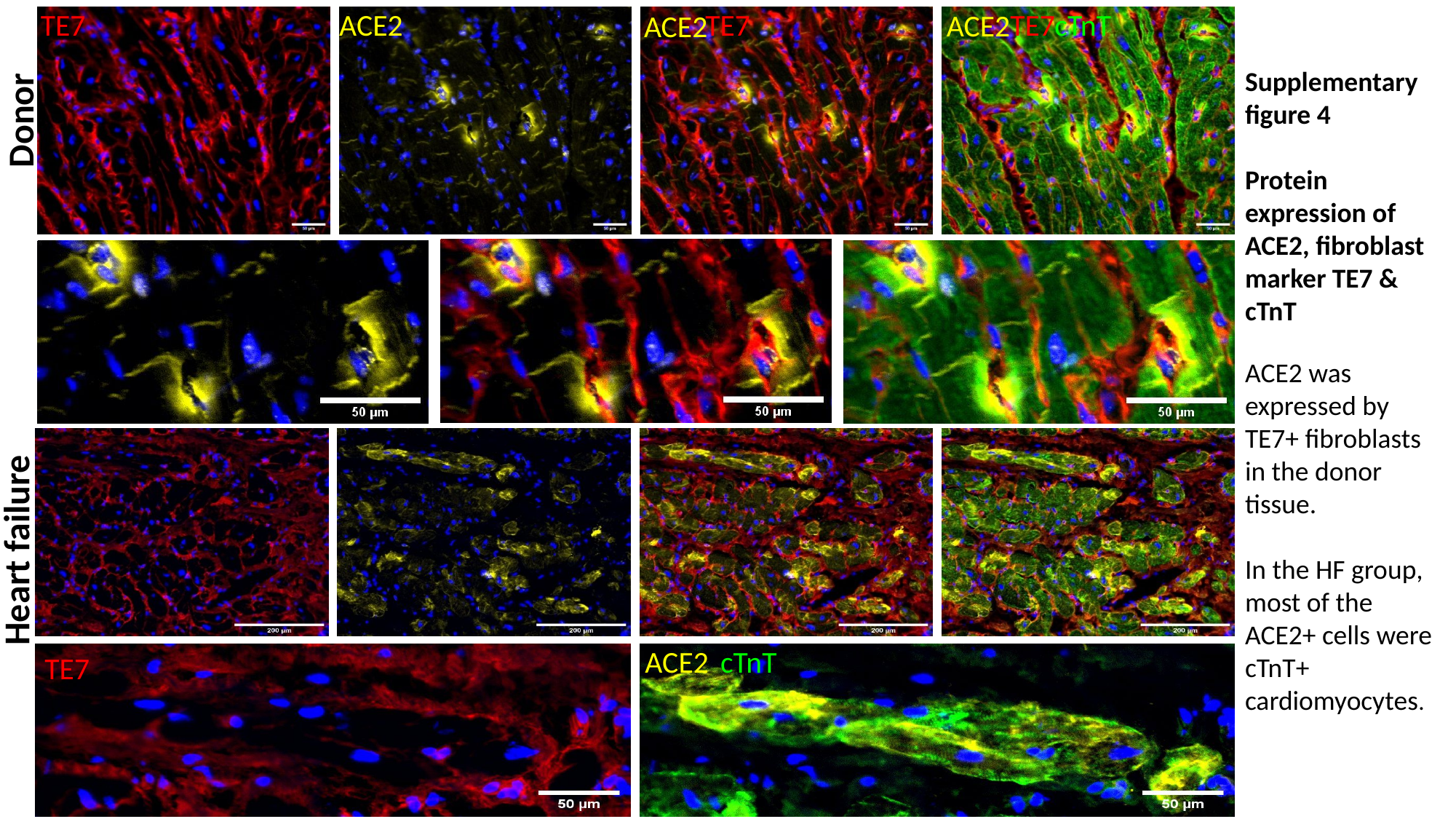

TE7
cTnT
ACE2
TE7
TE7
ACE2
ACE2
Donor
Supplementary figure 4
Protein expression of ACE2, fibroblast marker TE7 & cTnT
ACE2 was expressed by TE7+ fibroblasts in the donor tissue.
In the HF group, most of the ACE2+ cells were cTnT+ cardiomyocytes.
Heart failure
ACE2
cTnT
TE7

## Slide 6
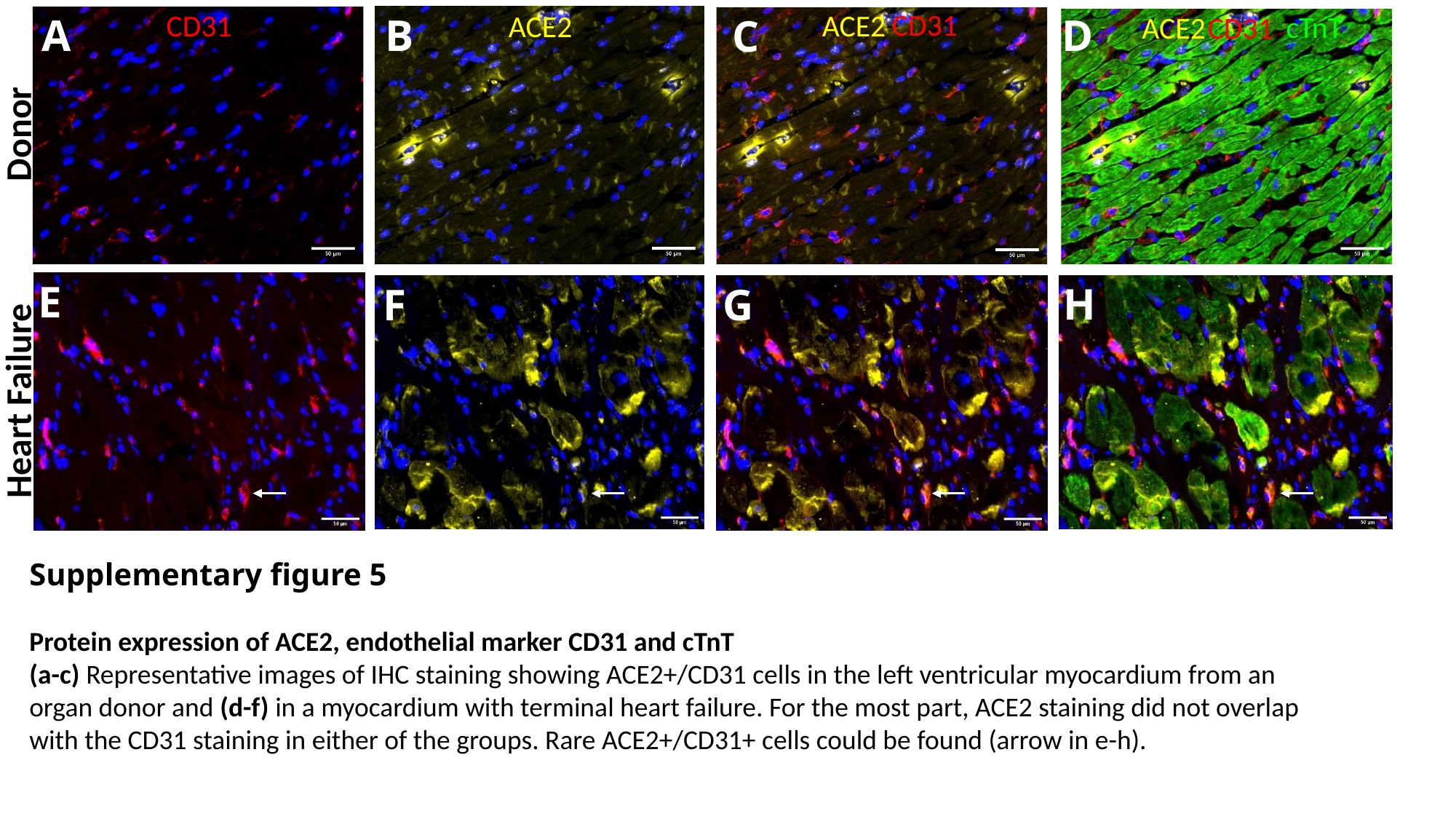

CD31
ACE2
ACE2
cTnT
ACE2
CD31
CD31
A
B
D
C
Donor
E
H
F
G
Heart Failure
Supplementary figure 5
Protein expression of ACE2, endothelial marker CD31 and cTnT
(a-c) Representative images of IHC staining showing ACE2+/CD31 cells in the left ventricular myocardium from an organ donor and (d-f) in a myocardium with terminal heart failure. For the most part, ACE2 staining did not overlap with the CD31 staining in either of the groups. Rare ACE2+/CD31+ cells could be found (arrow in e-h).
